# Opportunities and Barriers to Rural Telerobotic Surgical Health Care in 2021: Report and Research Agenda from a Stakeholder Workshop

**DOI:** 10.1089/tmj.2021.0378

**Published:** 2022-07-04

**Authors:** Ryan N. Hansen, Basil Matthew Saour, Brian Serafini, Blake Hannaford, Lanu Kim, Takayoshi Kohno, Ryan James, Wayne Monsky, Stephen P. Seslar

**Affiliations:** ^1^ The Comparative Health Outcomes, Policy, and Economics Institute, University of Washington, Seattle, Washington, USA.; ^2^ Advocate Heart Institute, Elgin, Illinois, USA.; ^3^ Sociology Department, University of Washington, Seattle, Washington, USA.; ^4^ Department of Electrical Engineering, University of Washington, Seattle, Washington, USA.; ^5^ School of Humanities and Social Sciences, Korea Advance Institute of Science and Technology (KAIST), Daejeon, South Korea.; ^6^ Paul G. Allen School of Computer Science & Engineering University of Washington, Seattle, Washington, USA.; ^7^ Telerobotics, LLC, Seattle, Washington, USA.; ^8^ Department of Radiology and University of Washington, Seattle, Washington, USA.; ^9^ Department of Cardiology, University of Washington, Seattle, Washington, USA.

**Keywords:** telemedicine, telehealth, telesurgery, telerobotics, rural, health care delivery, stakeholders

## Abstract

**Background:**

There are well-recognized challenges to delivering specialty health care in rural settings. These challenges are particularly evident for specialized surgical health care due to the lack of trained operators in rural communities. Telerobotic surgery could have a significant impact on the rural–urban health care gap, but thus far, the promise of this method of health care delivery has gone unrealized. With the increasing adoption of telehealth over the past year, along with the maturation of telecommunication and robotic technologies over the past 2 decades, a reappraisal of the opportunities and barriers to widespread implementation of telerobotic surgery is warranted. Here we report the outcome of a rural telerobotic stakeholder workshop to explore modern-day issues critical to the advancement of telerobotic surgical health care.

**Materials and Methods:**

We assembled a multidisciplinary stakeholder panel to participate in a 2-day Rural Telerobotic Surgery Stakeholder Workshop. Participants had diverse expertise, including specialty surgeons, technology experts, and representatives of the broader telerobotic health care ecosystem, including economists, lawyers, regulatory consultants, public health advocates, rural hospital administrators, nurses, and payers. The research team reviewed transcripts from the workshop with themes identified and research questions generated based on stakeholder comments and feedback.

**Results:**

Stakeholder discussions fell into four general themes, including (1) operating room team interactions, (2) education and training, (3) network and security, and (4) economic issues. The research team then identified several research questions within each of these themes and provided specific research strategies to address these questions.

**Conclusions:**

There are still important unanswered questions regarding the implementation and adoption of rural telerobotic surgery. Based on stakeholder feedback, we have developed a research agenda along with suggested strategies to address outstanding research questions. The successful execution of these research opportunities will fill critical gaps in our understanding of how to advance the widespread adoption of rural telerobotic health care.

## Introduction

There are well-recognized challenges to delivering health care in rural settings.^1–4^ These challenges are particularly evident for surgical procedures due to the lack of local access to general^5^ and specialty surgeons in most rural communities. Rural telerobotic surgery allows a solo urban-based surgeon to perform an operation on a patient in a rural setting by controlling a surgical robot over telecommunication technologies (e.g., fiberoptic cable and cellular telephone). The promise of telesurgery has been evident since the 2001 “Lindbergh Operation,” when the New York-based Dr. Jaques Marescaux operated on a patient in Strasbourg, France, using a Zeus robot connected to a transatlantic fiberoptic cable.^6^ Despite this promising start, telerobotic operations have been limited to isolated demonstration cases^7^ and a small clinical case series.^8,9^ However, with the increased adoption of telehealth over the past year, along with the maturation of telecommunication and robotic technologies over the past two decades, a reappraisal of the opportunities and barriers to widespread implementation of telerobotic surgery is warranted.

This report summarizes the outcomes of a 2-day international stakeholder workshop hosted online by our telerobotic research team at the University of Washington. The workshop's purpose was to assemble a multidisciplinary stakeholder panel to explore modern-day opportunities and barriers to the advancement of telerobotic surgical health care. Though we encouraged stakeholders to explore all aspects of telerobotic surgical health care, we did not directly address patient-related issues (patient adoption, informed consent, ethic, etc.), but rather emphasized aspects relevant to the emerging environment for future work and workers as part of the National Science Foundation's (NSF) core research focus, “Future Work at the Human-Technology Frontier.” As a result, several themes emerged during the workshop, providing us with a roadmap for developing a research agenda to facilitate the future widespread implementation of telerobotic health care.

## Methods

### STAKEHOLDER RECRUITMENT

We identified key stakeholders with relevant expertise in engineering, telecommunications, telehealth, health care economics, legal, sociologic, and regulatory issues through academic literature review, internet search, and colleague networks. Potential stakeholder participants were sent an invitation summarizing the workshop's goals, itinerary, and a registration link. Stakeholders in the network of one or more of the research team members were also directly contacted by email or telephone.

There were 24 participants (not including the research team) over the two sessions. Stakeholder domain expertise included surgeons specializing in colorectal surgery, electrophysiology, otolaryngology, and urology; technology experts representing computer science, engineering, robotics, and digital health; and health care experts trained in the fields of economics, law, public health, hospital administration, nursing, and health insurance. Most stakeholders participated in multiple breakout sessions on both days.

### THE WORKSHOP

Our University of Washington research team hosted this Telerobotic Stakeholder Workshop on January 12 and 19, 2021, through Zoom. The online format (required by COVID-19–related travel restrictions) allowed us to recruit additional international attendees. The workshop was partitioned into two 2-h meetings 1 week apart (*Table 1*). We divided each meeting day into two 20-min presentations, each followed by two 20-min breakout sessions. The presentations provided a shared context on topics integral to the implementation of telesurgery (*Table 1*). For the first breakout session, we preassigned stakeholders to a particular breakout room to encourage discussion *across* domains of expertise. In the second breakout session, we assigned specific topics to each room and allowed stakeholders to choose which room to join. A member of the research team moderated each room. Breakout room subject labels included regulatory/legal/industry/ethical, technical, reimbursement/health care economics, and clinical care/health care workflow/patient adoption.

**Table 1.** Stakeholder Workshop Format

	DAY #1	DAY #2
Welcome/overview	Steve Seslar—moderator	Steve Seslar—moderator
Presentation #1	“Understanding and Promoting Telehealth: Defining the Role for Research and Evaluation” presented by Annette M. Totten, PhD	“Telerobotic Surgery: A Law and Policy Roadmap” presented by Ryan Calo, JD, and “The Impact of COVID on Telehealth and Remote Monitoring Technologies” by Shwetak Patel, PhD
Whole group	Discussion	Discussion
Breakout #1	Participants assigned to rooms	Participants Assigned to Rooms
Breakout #2	Topics assigned to rooms	Topics Assigned to Rooms
Presentation #2	“Perspectives on Medical Education and Rural Surgical Care” copresented by Anjali Kumar, MD and Jamie Litvack, MD	“Current State of the Technology for Telerobotic Healthcare” by Greg Fischer, PhD
Whole group	Discussion	Discussion
Breakout #1	Participants assigned to rooms	Participants assigned to rooms
Breakout #2	Topics assigned to rooms	Topics assigned to rooms

### ANALYSIS

All portions of the 2-day stakeholder workshop, including the breakout sessions, were recorded with permission from the stakeholders. Recordings were converted into written transcripts by a third party vendor (REV.com). The content was first tagged and categorized using Atlas.ti (Supplementary Data), a qualitative data analysis tool that allows researchers to quickly analyze large amounts of textual information. All transcripts were then independently reviewed by two members of the research team (R.N.H. and B.M.S.) and statements made by the participants were organized into themes (e.g., credentialing and certification, technology, clinical, and economic) by all members of the research team through a consensus conference. As a group, we then converted stakeholder statements within each theme into research questions. Finally, we suggested possible research strategies that could be used to address specific research questions.

## Results

Overall, we found that the breakout room discussions fell into four general themes, including (1) operating room (OR) team interactions, (2) education and training, (3) network and security, and (4) economic issues.

### THEME 1: OR TEAM TRUST, LEADERSHIP, AND COMMUNICATION

The impact of trust, leadership, and communication on OR team performance during conventional and in-the-room robotic surgical procedures is well recognized. A surgical team typically has an extrinsic hierarchy^10,11^ that is defined in terms of the formal titles of the team members (e.g., surgeon, anesthesiologist, technician, and circulating nurse). Nonetheless, these same individuals often interact on the basis of an informal social construct based on the nature of the relationships between the team members. Like all relationships, the foundation of these informal interactions is based on trust. Team dynamics in the OR have been well studied, principally in the context of conflict resolution.^12–15^ More recently, researchers have begun to investigate the impact of “in-the-room” robotic surgery, in which the surgeon is displaced by the robot from the OR team around the patient on the OR table but remains in the room or an adjacent room using a tethered robotic controller. These studies have found that even this subtle displacement of the surgeon from the team causes meaningful effects on the team dynamics.^16–19^ It was, therefore, no surprise that there was a great deal of discussion about the potential impact of geographic separation of the operator on surgical team dynamics (*Table 2*). Core concepts of trust and teamwork among team members and between the team and the remote operator were explored in these conversations. Stakeholders expressed concerns that the usual OR teamwork dynamics would be disrupted by the surgeon being remote. They then offered suggestions on how to restore a cohesive team atmosphere, such as encouraging informal remote discussions between OR staff and the distant operator before starting the procedure to build team rapport. Ensuring ongoing interactions between remote operators and local physicians was also felt to be necessary to establish trust and connection.

**Table 2.** Representative Quotes and Research Questions Related to Team Trust, Leadership, and Communication

Representative quotes	1. “So much of a good surgical team is about relationships with the other team members.” 2. “Who is responsible when things go wrong?” 3. “Who takes over if we cannot communicate with the remote operator?”

SUBTHEMES	RESEARCH QUESTIONS
2.1. Team members	2.1a. In a “hub-and-spoke” model, a single operator will interact with multiple OR teams from different hospitals. How does the frequent change in OR team composition impact surgical team performance? 2.1b. Does telerobotic surgery lead to more or less stress for the surgeon? (both during and after a “learning curve”) 2.1c. How does telerobotic surgery impact the role of the first assistant? The scrub practitioner? 2.1d. How does OR team experience level in conventional procedures impact performance during telerobotic operations?
2.2. Team hierarchy	2.2a. How does the geographical separation of the surgeon impact their leadership (hierarchy) among the OR team; in the event of a crisis?
	2.2b. In the event of complete network failure and loss of contact with the remote operator, who assumes leadership and responsibility for the patient's well-being?
2.3. Team communication	2.3a. How does the situational awareness of the surgeon and communication with the team change in telerobotic compared with conventional operations? 2.3b. How can OR team behavior be modified to impact the remote operator's situational awareness? 2.3c. How can technology impact the remote operator's situational awareness?

OR, operating room.

One prominent concern was what happens when things go wrong during an operation. It was acknowledged that in times of crisis, the surgeon often functions as the team leader. In the context of telerobotic operations, the surgeon's ability to fulfill that role was called into question. Stakeholders considered the hierarchy of surgical teams during telerobotic operations. They discussed whether the remote surgeon would be the team leader in this situation. Furthermore, they reflected on who would ultimately be responsible for the outcome of the case.

### THEME 2: EDUCATION, TRAINING, AND CERTIFICATION

Rural hospital OR staff will initially be inexperienced in specialized surgerical procedures and both OR staff and operators will initially be unfamiliar with telesurgery. In this context, hospitals are challenged with developing both the training methods and the tools to measure the effectiveness of that training for telerobotic operations. Fortunately, there is an extensive literature from which to draw on for both robotic training^20,21^ and team training validation methods.^19^ Stakeholder discussions on education ranged from who to teach, what to teach, how to teach, and how long it would take. This topic also branched into credentialing—that is, how to decide when the surgeon and team are sufficiently trained to engage in clinical telerobotic operations (*Table 3*). Selection of which staff and members of the local OR team should be involved in telerobotic operations was also a topic of concern. Stakeholders asked whether all local rural OR team members should be trained in telerobotic procedures or should a select group of the OR staff choose to participate.

**Table 3.** Representative Quotes and Research Questions Related to Education, Training, and Certification

Representative quotes	“Education for everything from the physician, the nurse, to how to use the equipment to IT, is huge” “When should you train physicians to learn this system?” “How do you credential someone to do this? or do you even need to?” “Who has to understand how to fix the robot?”

SUBTHEMES	RESEARCH QUESTIONS
3.1. Team members	3.1a. Should everyone in the OR be cross-trained for telerobotic surgery or just specific OR team members?
	3.1b. Should the use of the telerobotic surgery be introduced to fellows in training? Early career MDs? Or mid-late career MDs?
3.2. Team credentialing	3.2a. What should the requirements be to determine whether the operator is competent in telerobotic surgery?
	3.2b. What should the requirements be to determine whether members of the OR staff are competent in telerobotic surgery?
3.3. Setup and maintenance	3.3a. Does the presence of a site-specific engineer alter the ability to safely and reliably perform the procedure?
3.4. Training	3.4a. Who should take ownership over training? Industry? Hospitals? A hybrid model? 3.4b. What are expected learning curve durations: (A) for each individual role on the OR team? (B) for the team as a whole? (C) for each team composed of specific individuals?

### THEME 3: NETWORK PERFORMANCE AND SECURITY

Historically, network latency and bandwidth were significant technical and economic impediments to moving telerobotic operations forward. Not surprisingly, reliance on internet network connections to perform telerobotic surgery remained a significant concern for many stakeholders (*Table 4*). It was an area identified by stakeholders in which basic information on network reliability for widespread telerobotic operation was lacking. Stakeholders wanted clarity not just on the chance of complete network failure but also the effects of transmission delays (latency) and degradation (jitter) on operator performance. Finally, concern was expressed regarding the security of the telerobotic system against cyber threats.

**Table 4.** Representative Quotes and Research Questions Related to Network Performance and Security

Representative quotes	“What happens when technology goes awry and there's a delay in response?” “How much network delay can be tolerated without affecting the procedure?” “How do you guarantee information is secure within the network?”

SUBTHEMES	RESEARCH QUESTIONS
4.1. Technology	4.1a. How will conventional in-the-room robotic equipment respond to issues like network delay?
4.2. Network performance	4.2a. At what frequency can we anticipate network delays or failures to compromise telerobotic surgical procedures?
	4.2b. What is the maximum acceptable delay in network responsiveness?
	4.2d. Do delays during different phases of the procedure or in the transmission of specific types of information have varying impact on surgical performance
4.3. Network security	4.3a. How can the system be designed to prevent loss of privacy?
	4.3b. How can one verify the system is able to defend against man-in-the-middle cyberattacks?
	4.3c. How can one verify that the person operating the robot is the correct surgeon, the robot is the correct robot, and the patient is the correct patient?

### THEME 4: ECONOMIC ISSUES

There was agreement from various stakeholders that telerobotic technology without an economically rational means to implement it would be fruitless (*Table 5*). This focus is in alignment with commonly cited implementation barriers to telehealth in general.^22^ Stakeholder discussions regarding cost focused on two major areas. First, stakeholders were concerned about upfront and maintenance costs for the technology to perform the telerobotic procedures in rural community hospitals. Second, they expressed a need for a broader cost-effectiveness analysis to better understand how specialty health care procedures, when offered by rural community hospitals, will financially impact insurance payors and rural health care more generally.

**Table 5.** Representative Quotes and Research Questions Related to Economic Issues

Representative quotes	“Who's going to pay for this education?” “What is the upfront startup cost for the robot?” “How many procedures are required to make this a cost-effective investment?”

SUBTHEMES	RESEARCH QUESTIONS
5.1. Training costs	5.1a. What are the anticipated costs of implementing a telerobotic training program for rural community hospital staff?
	5.1b. What are the anticipated costs of implementing a telerobotic training program for remote operators?
5.2. Equipment costs	5.2a. What are the anticipated capital, disposable, and maintenance costs for the telerobotic system?
5.3. Cost-effectiveness	5.3a. How many procedures need to be performed per month to make the system/service cost-effective?
5.4. Health insurer incentives	5.4. Where is the value from implementing coverage of telerobotic procedures realized for the payers of health care?

### RESEARCH STRATEGIES

Despite the relatively unexplored nature of telerobotic surgery, for each of the themes already outlined, there are a wealth of applicable and well-established methods that can be employed to answer the identified research questions outlined in Tables 2–5 (*Table 6*). For questions related to trust and communication among OR teams, researchers can draw from the extensive ethnographic workplace studies already performed for conventional and robot-assisted surgery. Similarly, methods in the field of implementation science such as the context, input, process, and project (CIPP) model can be used for developing and validating a medical training curriculum. Cost-effectiveness modeling to evaluate new medical treatment options is increasingly prevalent in today's cost-conscious health care environment. Finally, tools for network performance measurement and cybersecurity risk assessment have been well described.

**Table 6.** Selected Research Strategies and Applicable Questions

RESEARCH STRATEGY	APPLIES TO RESEARCH QUESTION	SAMPLE REFERENCE
Ethnographic and interview/survey-based study of established versus newly created OR teams performing simulated telerobotic surgery	2.1, 2.3	Pelikan et al. (2018)^17^ Randell et al. (2017)^19^
Catalog roles and behaviors into an ethogram to quantify OR behavior	2.2	Jones et al. (2016)^10^; (2018)^23^
Analysis of surgeon biometrics	2.1b	Ciraulo et al. (2020)^24^
Implementation science specifically related to adoption of new health care technologies	3.1–3.4	Grossi et al. (2021)^25^; Stone and Lane (2012)^26^
Simulation, mockup, and remote packet reflector networking studies	4.1, 4.2, 4.3	Sankaranarayanan and Hannaford (2008)^27^
Leverage multipath routing to mitigate network performance issues	4.1, 4.2	Tarique et al. (2009)^28^
Threat modeling and analysis of novel cybersecurity risks.	4.3	Checkoway et al. (2011)^29^; Koscher et al. (2010)^30^
Characterize the implementation costs of telerobotic surgery for rural hospitals	5.1–5.2	Totten et al. (2019)^31^; Reider-Demer et al. (2021)^32^
Demonstrate the economic viability of a telerobotic surgery service for a rural hospital and its urban surgeon(s)	5.3	Landaas et al. (2020)^33^
Estimate the impact of telerobotic surgery on rural health disparities	5.3	Ryskina et al. (2021)^34^

## Discussion

This study's primary outcome was developing a research agenda based on unanswered stakeholder-driven questions regarding the widespread implementation of telerobotic surgery in rural community hospitals. We intend to use this broad stakeholder feedback to develop a reseach and development roadmap for the collaborative multidisciplinary work needed to answer these important questions to facilitate the adoption of telerobotic health care.

### KEY OUTCOMES

This stakeholder workshop broadened our understanding of the significant current barriers to implementing telerobotic surgical health care in rural settings. In addition, it allowed us to develop a suggested research framework around them. This workshop also served to identify a group of individuals with diverse backgrounds that share an interest in advancing this cause. Although our research team will use this workshop's results to plan and prioritize our research strategy, we hope it will also serve as a guide to other research groups who share this mission. It should also be seen as an invitation to these groups to collaborate with us to achieve the relevant milestones.

### STRENGTHS AND LIMITATIONS

Our workshop had a broad representation of stakeholders in the future of telerobotic surgical health care. These participants grounded our discussions in the practical realities of current clinical practice, regulatory policies, reimbursement issues, and so on. Also, our stakeholders did not just raise issues, but they also provided strategies for addressing those issues guided by their domain expertise. Several of our stakeholder participants represented providers on the frontlines of rural health care who understand firsthand the challenges and opportunities afforded by rural telerobotic health care.

Because we intentionally focused our attention on the future of work and rural telerobotic health care workers, our panel lacked important representation from patients and other community groups. This omission could have prevented us from capturing critical research questions related to patients and their attitudes toward this form of health care delivery. Although we did have specialty surgeons on our stakeholder panel, we did not have representation from their professional societies. We also did not have representation from the Center for Medicare Services (CMS). Future stakeholder engagements should include constituents of these groups. Such inclusion will be critical for future widespread adoption.

Although we had international representation, most stakeholders were from the United States. This meeting, therefore, predominantly addresses rural telerobotic health care from that perspective. Although many issues we have identified may be universal, there will undoubtedly be unique challenges across each of the themes for different geographic regions that we did not consider here. Countries such as Canada, China, and India may be advancing telerobotic surgery more quickly than what has been achieved in the United States. Thus, along with unique challenges, there may be unexpected opportunities in those regions we have missed in our workshop. Another potential impact of teleorobotic surgery, expanding access to advanced in the developing world, deserves its own workshop.

Finally, this workshop represents just a snapshot in time. The challenge is to translate and operationalize the teachings from this effort and then reconvene similar groups as progress is made. Our goal is to foster research among collaborative groups performing convergent research to achieve a common goal: advancing the field of rural telerobotic surgical health care.

## Conclusions

The U.S. population is aging at an unprecedented rate.^10^ Each day, >10,000 people in the United States reach the age of 65 years.^1^ More concerning, the elderly, who are generally most in need of specialty health care, disproportionately reside in rural communities,^10,11^ making up ∼25% of the rural population in the United States. From a workforce perspective, rural communities struggle to hire and retain specialty surgeons, with more than half of rural counties in the United States having no surgeon whatsoever.^12^ Together, this shift in population dynamics, coupled with an inadequate surgical workforce, creates a clear and present need to develop a future workforce that can address the large and growing rural health care disparity.

The widespread adoption of telerobotic surgery could have a profoundly positive impact on the future of rural health care. The COVID-19 pandemic has created unprecedented acceptance of telehealth in general. Organizations such as the American Telemedicine Association (ATA) are providing a critical infrastructure to bring about policy changes necessary to accelerate the growth of this critical form of health care delivery. Equally important in adoption of telesurgery will be the leadership of professional societies such as the American College of Surgeons that have pioneered all manner of telesurgery including telementorship, teleproctoring, and telesurgical simulation.

This workshop directly addressed stakeholder-identified barriers to rural telerobotic health care and will help us pioneer the technologies most critical to overcome them. The successful execution of the research opportunities identified here will fill critical gaps in our understanding of how the surgeon's displacement from the OR impacts OR team performance. It will also help us understand how to train future workers for this new form of health care delivery. Guided by the research agenda presented here, we will enhance our understanding of the current rural community hospital network infrastructure and develop strategies to execute telerobotic surgery under such constraints. Finally, this study ensures that the technology developed will conform to the economic reality of rural health care delivery. We invite all interested parties to use the insights and research directions provided here to work with us to help telerobotic surgery fulfill its promise of bringing advanced surgical health care to patients living in the rural United States and austere regions around the world.

## Supplementary Material

Supplemental data
