# Supplementary material for: Opportunities and Barriers to Rural Telerobotic Surgical Health Care in 2021: Report and Research Agenda from a Stakeholder Workshop
Source: Telemed J E Health. 2022 Jul 4;28(7):1050–7. doi: 10.1089/tmj.2021.0378 (PMC9293678; doi:10.1089/tmj.2021.0378)
Supplement: Supplemental data [file Suppl_Data.pdf]

ATLAS.ti Report

NSF Workshop

Quotations grouped by Documents

Report created by Ryan Hansen on Feb 19, 2021

---

1 BS_day1_audio_only.pdf

13 Quotations:

1:1 p 2 in BS_day1_audio_only.pdf

Content:

> what happens when technology goes awry and there's a delay in response

1:2 p 2 in BS_day1_audio_only.pdf

Content:

> so much of a good surgical team is about relationships with the other team members

1:3 p 3 in BS_day1_audio_only.pdf

Coding:

○ trust

Content:

> trust

1:4 p 3 in BS_day1_audio_only.pdf

Coding:

○ collaborative

Content:

> collaborative

1:5 p 3 in BS_day1_audio_only.pdf

Coding:

○ mentorship

Content:

> mentorship

1:6 p 4 in BS_day1_audio_only.pdf

Content:

> fewer redo cases, fewer complications, shorter procedure time, something to show payers that it's worth investing in that second operator in that context.

1:7 p 4 in BS_day1_audio_only.pdf

Content:

> how much confidence is a patient have in the person that they communicate with online?

1:8 p 5 in BS_day1_audio_only.pdf

Content:

> I think that one of the big things is also going to be age

1:9 p 5 in BS_day1_audio_only.pdf

Content:

> I think that we might have to do something different as far as communication with the elders

1:10 p 5 in BS_day1_audio_only.pdf

Content:

> such a high value for staying in your community

1:11 p 6 in BS_day1_audio_only.pdf

Content:

> who's going to pay for this big education

1:12 p 7 in BS_day1_audio_only.pdf

Content:

> We just need the education and the resources and to know we will make do, but we want to know that there's people out there backing us up

1:13 p 9 in BS_day1_audio_only.pdf

Content:

> make sure that there is no increased risk of the safety side

2 BS_day2_ breakout room 2.1.pdf

2 Quotations:

2:1 p 6 in BS_day2_ breakout room 2.1.pdf

Content:

> he cognitive work that goes into just getting the systems up and running that are safe for patients is pretty high and that takes time

2:2 p 9 in BS_day2_ breakout room 2.1.pdf

Content:

> I think the outcomes and the commitment to the facility to have dedicated staff for robotics or a situation like we do any type of telerobotics is really important, and education from everything from the physician, the nurse, how to use the equipment to IT, which is huge. And then even central service to cleaning supplies.

3 LK_day1_audio_only.pdf

6 Quotations:

3:1 p 2 in LK_day1_audio_only.pdf

Content:

> I think education is going to be a massive undertaking with this

3:3 p 3 in LK_day1_audio_only.pdf

Coding:

- ○ drilling

Content:

> drilling

3:4 p 3 in LK_day1_audio_only.pdf

Content:

> I think the financial piece is huge for rural.

3:5 p 8 in LK_day1_audio_only.pdf

Content:

> it's key that their primary care provider is on board and can manage up

3:6 p 10 in LK_day1_audio_only.pdf

Content:

> sub tasks automation

3:7 p 11 in LK_day1_audio_only.pdf

Content:

> One of one of the challenges in robotics in general is keeping an up to date model of the anatomy as the surgery evolves

4 LK_day2_audio_only.pdf

4 Quotations:

4:1 p 13 in LK_day2_audio_only.pdf

Content:

> team dynamics

4:2 p 13 in LK_day2_audio_only.pdf

Content:

> social consequences of telerobotic healthcare

4:3 p 16 in LK_day2_audio_only.pdf

Content:

> spectrum of the degree of invasiveness of the intervention

4:4 p 22 in LK_day2_audio_only.pdf

Content:

> oom to investigating dedicated evaluation metrics to quantify those telerobotic procedures

---

7 RH_day1_Econ Breakout Audio.pdf

1 Quotations:

7:1 p 4 in RH_day1_Econ Breakout Audio.pdf

Content:

> would think that the cost, you would hope would be somewhat neutral or less perhaps, if you're able to gain the efficiencies

---

8 RJ_day1_audio_only.pdf

1 Quotations:

8:1 p 2 in RJ_day1_audio_only.pdf

Content:

> can you get a system like this to degrade gracefully as the network performance tails off in terms of digital throughput

9 RJ_day1_audio_only_1.pdf

1 Quotations:

9:1 p 2 in RJ_day1_audio_only_1.pdf

Content:

> who takes responsibility if an outcome isn't ideal?

---

10 RJ_day1_audio_only_2.pdf

2 Quotations:

10:1 p 3 in RJ_day1_audio_only_2.pdf

Content:

> alternative, or additional imaging modalities, that might be required to facilitate automation?

10:2 p 4 in RJ_day1_audio_only_2.pdf

Content:

> there's a big conversation in the surgeon community about repetitive injuries.

---

13 RJ_day2_audio_only_2.pdf

2 Quotations:

13:1 p 2 in RJ_day2_audio_only_2.pdf

Content:

> step-wise approach

13:2 p 2 in RJ_day2_audio_only_2.pdf

Content:

> how you would evaluate the added or reduction, the change in risk

14 sps_day1_audio_only.pdf

4 Quotations:

14:1 p 3 in sps_day1_audio_only.pdf

Content:

> COVID has actually helped us because now that insurance companies, although it's not very much money, it's not as much as seeing patients in the clinics, however, we are being reimbursed for the tele visits

14:2 p 6 in sps_day1_audio_only.pdf

Content:

> we are going to have to pay for this

14:3 p 6 in sps_day1_audio_only.pdf

Content:

> rural populations would love to have access to better care

14:4 p 10 in sps_day1_audio_only.pdf

Content:

> a community relationship that I think is different in a small town with a physician and done by telehealth

15 SPS_day2_audio_only.pdf

4 Quotations:

15:1 p 11 in SPS_day2_audio_only.pdf

Content:

> We'd have problems with our equipment just because they wouldn't clean them properly

15:2 p 12 in SPS_day2_audio_only.pdf

Content:

> fairly human resources, heavy at least at the stage we're at right now, but we really do find that that's required in order to resolve any difficulties that may raise in a timely manner, and also encourage utilization as well

15:3 p 14 in SPS_day2_audio_only.pdf

Content:

> people who do the reimbursements look at minimally invasive as one blanket thing, rather than all the nuances

15:4 p 14 in SPS_day2_audio_only.pdf

Content:

> A lot of people just feel like if they're going to get charged for an almost equivalent amount telehealth versus in-person, they'd rather just come in-person because there's more that a physician can do

16 TK_day2_audio_only.pdf

3 Quotations:

16:1 p 3 in TK_day2_audio_only.pdf

Content:

> we are used to sometimes doing things that are a little less comfortable but that do no harm

16:2 p 4 in TK_day2_audio_only.pdf

Content:

> the staff and the OR crew itself is excited

16:3 p 4 in TK_day2_audio_only.pdf

Content:

> we have some patients who refuse to leave, even if they would do better at another facility. So those patients, doing something is better than nothing if they insist on staying here.

17 TK_day2_audio_only_1.pdf

3 Quotations:

17:1 p 3 in TK_day2_audio_only_1.pdf

Content:

> situational awareness would just be really interesting

17:2 p 4 in TK_day2_audio_only_1.pdf

Content:

> studies that have shown that in the next 20 years we're going to have a huge depletion of surgeons and not enough surgeons to go around

17:3 p 4 in TK_day2_audio_only_1.pdf

Content:

> In electrophysiology, robotic surgeries can be much slower and tedious, but if you're in a situation where the catheter is very unstable, with robotics it's going to be done much faster.

18 TY_day1_audio_only.pdf

7 Quotations:

18:1 p 2 in TY_day1_audio_only.pdf

Content:

> the preparation might be additional cost that rural community need to pay attention. So, one thing is a training for the procedure, but also, another is training for maybe supporting environment to enabling the procedure in a rural area.

18:2 p 2 in TY_day1_audio_only.pdf

Content:

> we have to think about the instrument cost, and I think we need to think about the human resource cost.

18:3 p 2 in TY_day1_audio_only.pdf

Content:

> I worry about whether or not we'll get adequate staffing at these rural areas as well

18:4 p 3 in TY_day1_audio_only.pdf

Content:

> culture where issues come up and people would just not say anything, and we ended up having worst quality of care.

18:5 p 3 in TY_day1_audio_only.pdf

Content:

> we have to develop that communications necessary for each part, for the remote party and the local party to understand what is needed and to have all the resources needed to provide the proper surgical care.

18:6 p 5 in TY_day1_audio_only.pdf

Coding:

- ○ sub task automation

Content:

> sub task automation

18:7 p 6 in TY_day1_audio_only.pdf

Content:

> One of the challenges in robotics in general is keeping an up-to-date model of the anatomy as the surgery evolves.

19 TY_day1_audio_only_1.pdf

4 Quotations:

19:1 p 2 in TY_day1_audio_only_1.pdf

Content:

> afety definitely is one of the biggest barrier

19:2 p 3 in TY_day1_audio_only_1.pdf

Content:

> But a lot of the studies don't even acknowledge that there could be harms

19:3 p 6 in TY_day1_audio_only_1.pdf

Content:

> do a historical analysis in the past of harms that have arisen in prior surgeries that are not telerobotics, understanding what would have happened in that exact situation if telerobotics was used

19:4 p 7 in TY_day1_audio_only_1.pdf

Content:

> To have a senior surgeon from a ... who's done a rare procedure from across the world actually being able to weigh in on something like tha
